# Cryopreservation as a Key Element in the Successful Delivery of Cell-Based Therapies—A Review

**DOI:** 10.3389/fmed.2020.592242

**Published:** 2020-11-26

**Authors:** Julie Meneghel, Peter Kilbride, G. John Morris

**Affiliations:** Asymptote, Cytiva, Danaher Corporation, Cambridge, United Kingdom

**Keywords:** cell therapy, cryochain optimization, cryopreservation, freezing, cryogenic storage, cryogenic transport, thawing

## Abstract

Cryopreservation is a key enabling technology in regenerative medicine that provides stable and secure extended cell storage for primary tissue isolates and constructs and prepared cell preparations. The essential detail of the process as it can be applied to cell-based therapies is set out in this review, covering tissue and cell isolation, cryoprotection, cooling and freezing, frozen storage and transport, thawing, and recovery. The aim is to provide clinical scientists with an overview of the benefits and difficulties associated with cryopreservation to assist them with problem resolution in their routine work, or to enable them to consider future involvement in cryopreservative procedures. It is also intended to facilitate networking between clinicians and cryo-researchers to review difficulties and problems to advance protocol optimization and innovative design.

## Introduction

For a successful, clinical outcome of cell therapy (CT), the timely delivery of consistently reliable and effective cell materials to the point of receipt by the patient is critical. Significant difficulties arise when the required point of use is separated by distance and, increasingly commonly, by time from the facilities where the cells were isolated and prepared. Short-term storage, typically 2–4 days at 4°C, may be appropriate, in some instances, to alleviate such difficulties e.g., for bone marrow-derived mesenchymal stem cells (1), peripheral blood stem cells (2), pluripotent stem cell-derived cardiomyocytes (3), alginate-encapsulated adipose-derived stem cells (4), and hepatocytes (5). However, any greater extension of effective shelf life at even this lowered temperature will not be possible as metabolic decline and disruption will lead rapidly, and eventually, to total loss of cell viability. A long-term, practical solution to this difficulty lies in successful cryopreservation that offers secure, stable storage at temperatures below −130°C where metabolic change will not occur. The essential elements of the cryopreservation process as it can support cell therapy are outlined below, with a more detailed treatment of freezing and the science underlying cryopreservation available from reviews at a more fundamental level (6–13).

When effective procedures for long-term storage and transport are linked together (a cryochain) then timing of delivery of a therapy to the patient can be precisely controlled to secure the required clinical objectives (14–23). Additionally, with these extended storage times, cryopreservation provides clear benefits for cell banks and research collections of cells of ongoing value in regenerative medicine (23–25). Cryopreservation is also well-established as part of hematopoietic stem cell (HSC) transplantation, with over 47,000 procedures carried out in Europe in 2018, and has gained a specific importance in a context of global pandemic, where recommendations from transplant networks worldwide now include the cryopreservation of all products intended for allogeneic transplantation (13, 26–28).

For cryopreservation to be used with optimal success it is essential that the entire protocol of freezing, storage, and thawing procedures is carried out in the precisely required and prescribed manner. The team concerned with freezing must, therefore, be appropriately equipped, experienced and informed to be able to preserve the maximum viability in the frozen samples. The team responsible for the recovery of this viability, by thawing, need not be specialized in the earlier stages of cryopreservation but must be appropriately skilled and knowledgeable as to the detail of what is required for the best outcomes of their efforts (29). This is critical for, if the cryopreservation process results in sub-optimal cell recovery, it may not be possible to provide another treatment for further therapy.

**Figure 1:**
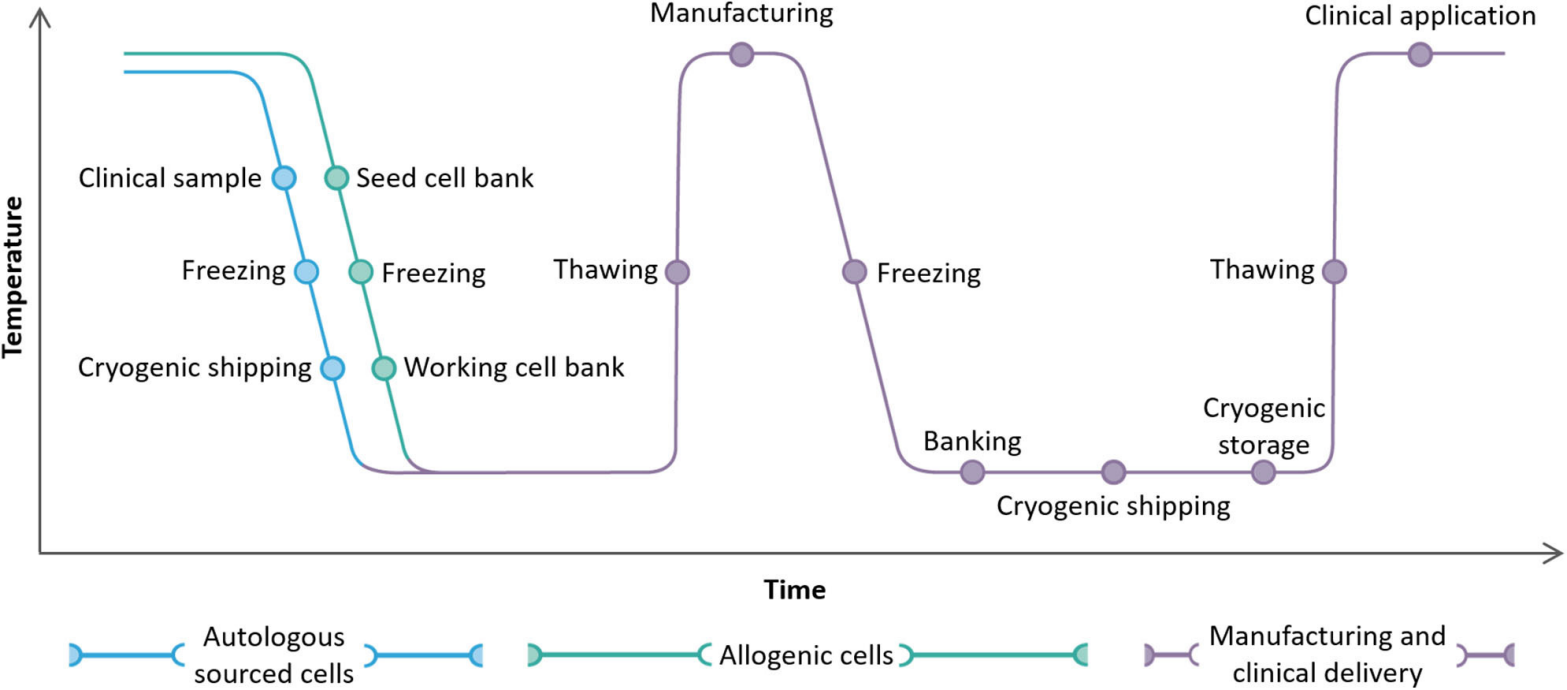
A complete cryochain for a cell therapy product from collection from a patient (autologous route, blue) or a healthy donor (allogeneic route, green) to manufacture and clinical delivery. Two cycles of freezing and thawing, cryogenic storage, and cryogenic shipping are illustrated.

Cryopreservation will provide additional benefits when used twice in the process chain from tissue isolation to delivery to the patient (Figure 1). An original isolate e.g., from biopsy or apheresis, can be stabilized and stored by cryopreservation until the appropriate time for further growth and/or manipulation to produce the final, therapeutic product. This final product can then, in turn, be cryopreserved and stored until the appropriate time and place for clinical delivery (8, 30, 31).

When larger quantities of tissue are being prepared for clinical use cryopreservation provides advantages over conventional short-term storage, or “just in time” manufacture, as it provides the capability to make large quantities of material available for subsequent use at some as yet unknown, point. Other advantages include:

- The availability of preparations of consistent quality from secure storage
- Flexibility and predictability of delivery for clinical scheduling.
- Global availability with transport not being time-critical
- Less product wastage, with reduced costs

In research environments, the key parameters for successful cryopreservation include effective cooling, thawing, ice nucleation strategies, and correct use of cryoprotectants. These remain critical when moving to clinical samples, but where cryopreserved CT products are employed there are, typically, regulatory issues that also have to be accommodated, in addition to safety precautions which must always be taken with clinical samples (32). For example, therapeutic materials provided as cells for parenteral application may be viewed as medicines and relevant, regulatory conditions could include providing evidence for:

- Minimized potential for contamination of the sample.
- Hermetically sealed samples.
- Reproducibility—all samples having the same viability and efficacy on thawing.
- Traceability throughout the cold chain and up to delivery to the patient.

An efficiently managed, secure, and monitored cryochain will meet all of these requirements.

It is important that the conditions necessary to ensure optimized cryopreservation and an effective cryochain are considered at an early stage in the development of any specific cell therapy. Practical details of the cryopreservation processes should be built-in as an early consideration as later redesign of, for example, sample containers or storage and transportation options can be difficult, time-consuming, and costly to implement. The freezing protocol may also be adversely affected by such alterations. It is, therefore, essential when developing a cryopreservation protocol to consider the practical requirements in reverse, that is from the point of delivery to the patient back to the acquisition and preparation of the source material. The mode of delivery to the patient will, for example, affect the type and size of the container cells are frozen in. In turn, this will influence the design of the cooling protocol and the requirements for handling, storage, shipping, and thawing. To avoid any bottleneck effect that cryopreservation may have on the timely delivery of the completed therapy, a holistic view of the cryochain should be kept in mind (Figure 1).

In this review the future, strategic steps toward delivery of effective, cryopreserved regenerative medicine products will be addressed, with a particular focus on T cell and other apheresis-based therapies. The necessary, key parameters, such as cryoprotectant use, cooling rate, ice nucleation, and other product-related parameters (volume, cell density, cryopreservation vessel etc.) which must be appropriately defined and controlled for the successful cryopreservation of mammalian cells for cell therapy are considered in appropriate detail. The critical storage, shipping, and thawing conditions will be considered, and future steps toward delivery of regenerative medicine products will be addressed.

The review also deals with those aspects of cryopreservation directly relevant to the clinical situation, from initial tissue isolation to the delivery of recovered cells to the patient. It will outline the steps that need to be taken to successfully modify an existing cryopreservation protocol. Working through such a process may not be the role of the clinical team concerned but an understanding of the process will support discussions with cryopreservation specialists working in research and development. In this regard an outline, practical guide to aid in the development of a successful cryopreservation protocol is provided (see Supplementary Material). The practical steps necessary to modify an existing cryopreservation protocol or devise a new one are essentially the same.

The presented material largely covers the delivery of cell-based therapies in regenerative medicine, cryopreserved in larger volumes in commercially available “cryobags.” Their processing will have used the widely published and adopted “slow cooling” cryopreservation protocols (15, 33). Vitrification-based approaches are not included as, currently, these are only effective for small volumes of cells, typically in reproductive medicine (34–36). The technique can be successful for mammalian gametes and embryos, but significant technical issues are involved in scaling up to larger tissue pieces and bulk cell suspensions (25, 37, 38). However, it may be of more immediate value for tissue banking of stem cells, where a low number of samples of small volume are required (39).

## Sourcing Cell Samples

The ability to secure and stabilize specific cell types, e.g., T cells from apheresis or cells taken by biopsy, is a critical first step in the development of successful cell therapies for regenerative medicine (11, 15, 23, 24, 40). Inevitably, these isolated, biological samples suffer increasing damage the longer they remain without physiological support *in vitro*, resulting in eventual cell death. Maintaining the samples on ice or with non-frozen refrigeration can provide a limited storage window (41) but the only way to preserve levels of viability and avoid progressive decline in cellular survival for longer timeframes is to stabilize the tissue by cryopreservation. If this procedure is applied as soon as possible after the initial tissue isolation (29, 42) or re-stimulation (43), then any loss of viability and/or function will be minimized and long-term storage extending into decades can be secured (44–48). Tissue biopsies such as melanomas can be stabilized and stored prior to extraction of tissue infiltrating lymphocytes (TIL) for expansion and transfer back to the patient (40). Biopsy samples of limited size (c. 1–3mm^3^, up to 1 mm in thickness) are likely to be suitable for immediate cryopreservation. Samples of ovarian or testicular tissue destined for subsequent autologous transplantation can be stabilized by cryopreservation and then stored until suitable arrangements for the transplantation can be made (35, 49–55). Larger tissue pieces may require further dissection or a level of enzymic digestion prior to preservation (56–61). As freezing technology progresses then larger pieces of tissue, and organs e.g., derived from liver or ovaries will become subjects of increasing interest with a view to their cryopreservation (62–65).

## The Freezing Process

To utilize cryopreserved, therapeutic products effectively, and to contribute to any necessary process refinement, it is important that the teams involved from sample preparation to delivery to the patient share an understanding of the freezing process. Similarly, researchers involved in developing new and innovative protocols for cryopreservation in regenerative medicine need a strong understanding of the principles that underpin the process.

The following descriptions outline the supporting principles of cryopreservation as they apply to the bulk cell suspensions that are the focus of this review.

### Cell Damage and the Role of Cryoprotectants

**Figure 2:**
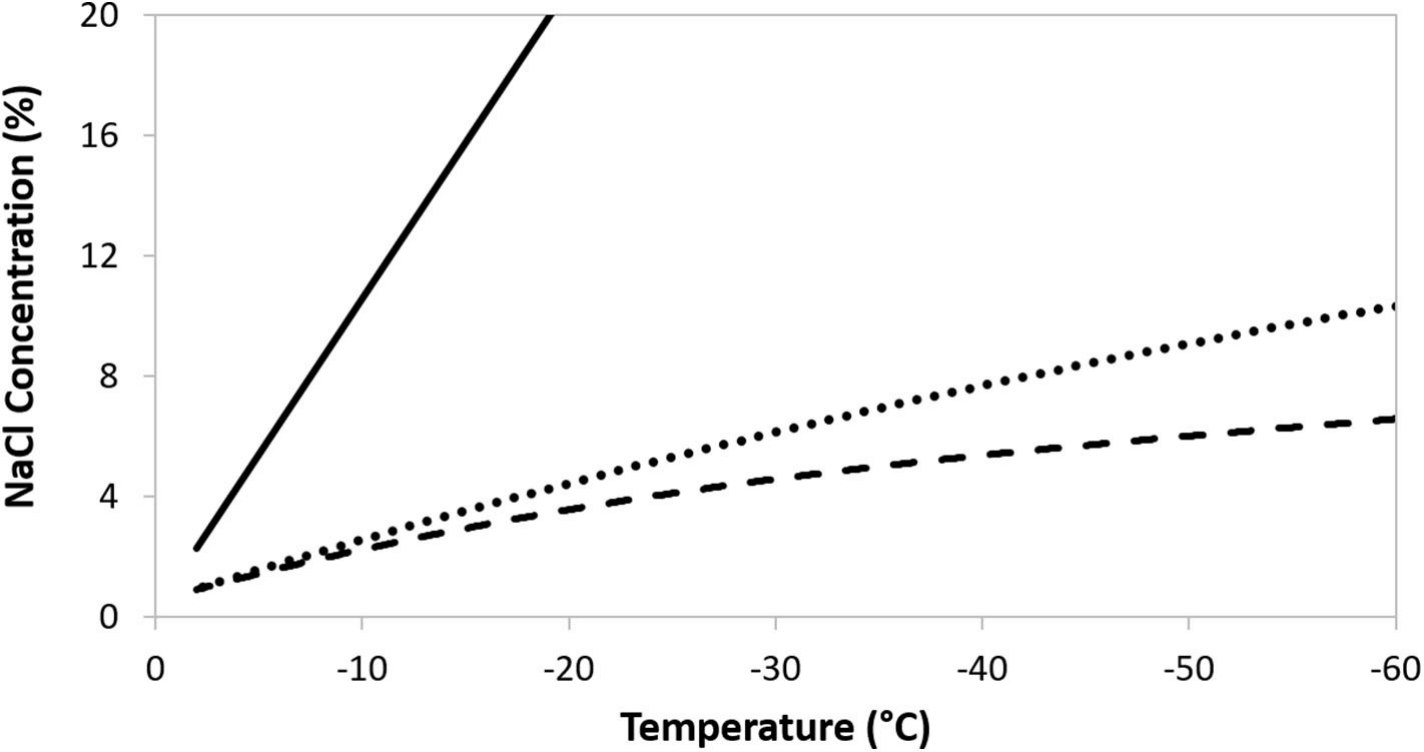
The increase in ionic concentration following freezing of a 300 mOsm NaCl solution (solid line), or a 300 mOsm NaCl solution containing DMSO at 5% w/v (dotted line) and 10% w/v (dashed line).

Cryopreservation can bring about a number of cellular injuries, potentially leading to deleterious changes in cell morphology, characteristics (e.g., adhesion, cell surface markers), metabolic activity (e.g., proliferation ability, potency), function (e.g., immunomodulation), and to cell death (6, 66). Temperature decrease can be responsible for triggering specific stress response pathways (7, 11) and can activate apoptotic and necrotic pathways after thawing (67). Significant cell injury can develop in response to the stressful changes that occur in the frozen sample due to ice crystal formation. As cooling progresses, ice initiates first in the suspending extracellular medium. The crystalline structure of the ice excludes solutes and so their concentration increases in the residual, unfrozen suspending liquid. A direct consequence is an increase in osmolarity of the suspending medium, imposing osmotic stresses on the cells (Figure 2). As temperature continues to fall the ice fraction increases, and with it the extracellular osmolarity. The resulting osmotic stress can cause severe injuries and is a major cause of viability loss during attempts at cryopreservation (7, 68, 69). Intracellular ice formation may also occur, which is a leading cause of cell death (6, 70–72).

To minimize the cell damage that arises, largely, from such solute effects it is essential to use added, cryoprotectant, compounds. These compounds do not ionize in aqueous solutions and, at low temperatures, have a relatively low toxicity to the cells (73). Added cryoprotectants are also excluded from ice crystals and have the effect of maintaining an enlarged, unfrozen extracellular fraction at any given temperature, thereby reducing the osmotic effect of the concentrating solutes (7, 74). In turn, this can significantly reduce osmotic injury. For illustration, the solute concentration in a sample of 300 mOsm NaCl during freezing is shown in Figure 2, with the traces plotting the increasing concentration of NaCl in the residual, unfrozen solution. As the temperature decreases the ice fraction increases, excluding NaCl from the crystals, and so the solute concentration in the residual solution rises. The ameliorating effect of increasing concentrations of the cryoprotectant DMSO are evident. Non-penetrating, extracellular solutes will assist cell dehydration to limit the probability of intracellular ice formation and can interact with, and stabilize, the limiting cell membrane (7). Additional, specific mechanisms of protection conferred by cryoprotectants will depend on their capacity to diffuse through the outer cell membrane. By increasing the intracellular osmotic concentration, permeating protectants will decrease the likelihood of ice forming inside cells, as well as limiting the extent of cell, and organelle, dehydration. Mixtures of permeable and non-permeable cryoprotectants can be used to provide a synergistic effect, but care has to be taken in their choice as any beneficial impact on osmotic stress may be offset by toxicity to the cells in question (7, 75). Toxicity may be reduced by ensuring that exposure to cryoprotectant is limited to suitable low temperature e.g., a refrigerator or ice bath (4°C) (73, 76, 77) and that the pre-freeze exposure time is minimized (29, 78). The most widely used, permeating protectant is dimethyl sulphoxide (DMSO) (15, 66). Glycerol and propanediol may also be encountered, although the former is more commonly used with erythrocytes (79, 80) and sperm cells (81, 82), while the latter is usually a component of vitrification solutions (83). Cell membrane permeability to the commonly used, permeable cryoprotectants will be c. 100–1,000 times lower than to water (68), so it is essential to include an adequate incubation time in the cryoprotectant to allow for equilibration of protectant between the extra- and intracellular compartments. An understanding of toxicity effects is critical for an optimal incubation period. Hydroxyethyl starch and oligosaccharides are common, non-permeating examples (7, 84, 85).

It is important to ensure that any cryoprotectant being considered has supporting evidence to show it can be used clinically e.g., approved to be transfused or injected into a patient. DMSO, for example, is a very effective cryoprotectant and many of the alternatives that have been developed are less so, especially when working with cells particularly sensitive to freezing and thawing (86). It has been used as a carrier solution in injections for many years and infused along with stem cells from bone marrow transplants (9, 66). The most commonly used concentration of DMSO is 10% v/v, as with stem cells (9, 66). A clear trend toward its reduction down to 5% v/v is apparent, in combination, or without, the inclusion of non-permeable CPAs, to both improve cell recovery post-thaw and reduce the amount of DMSO infused to patients (83, 87–91).

Currently, there is a developing discussion about the clinically appropriate use of DMSO for CTs and, whilst there have been very few contraindications for DMSO in transfusions of adult stem cells (92–94), there are questions about its suitability if the intention is to implant the cells directly into localized environments such as the brain or bone (95–99). Alternative cryoprotectants may be identified that are successful during research and development, and must be rigorously assessed on their safety for transfusion into a patient before taking the final steps toward a completed therapy.

### Sample Containers for Cryopreservation

The choice of appropriate sample container for cryopreservation depends upon the end use of the cell preparation, once recovered. For research purposes, small volumes of cell suspension are likely to be adequate and small sterile cryovials (typically 1–10 mL) can be the container of choice (23, 100). However, for therapeutic delivery, containers must be hermetically sealed to prevent the risk of contamination and for this reason alone research cryovials are not appropriate (15). The potential for contamination of unsealed CT samples by liquid nitrogen (LN)-borne contaminants (101) or other contaminants in the cryochain (102–104) means that samples for parenteral administration (injecting directly into the body) must be processed in hermetically sealed containers (105).

It should be noted that, to maintain strength at low temperatures (106), many vials designed specifically for medical use have thicker walls than standard, research laboratory cryovials. The material used to manufacture them may also have low thermal conductivity, and so care must be taken to ensure that the rate of temperature reduction in the vial reflects accurately the protocol entered into the programmable freezer (107). This can be achieved by inserting a recording temperature probe into a dummy sample during the cooling procedure. This becomes particularly relevant when the quality control sample is contained in a medical vial, with the bulk material of the preparation in a cryobag.

Many cell treatments require relatively large volumes (>100 mL) of suspended cells and these are held in specifically designed cryobags for cryopreservation. They are, of necessity, sterile and can be hermetically sealed before processing. In many instances they are cryopreserved within an additional, outer wrapping to guard against mechanical damage that might compromise the structure of the container, with risks of material loss and compromised sterility (15). Both the cryobag and the overwrap bag have relatively thin walls and the presence of any noticeable amount of air—a good thermal insulator—between them should be removed to ensure the temperature of the cell preparation is closely aligned with the cooling rate generated by the freezer. Instead of, or in addition to, the overwrap bag a metallic casing may also be used, which provides the additional advantages of insuring that the bag is frozen flat, thereby reducing any risks of breaking at cryogenic temperatures. More importantly, the casing has excellent heat-conducting properties to ensure a more consistent, homogeneous thermal profile across the length, and thickness of bags during freezing, which is essential to ensure homogeneous cell recovery between preparations.

Appropriate cryocontainers would include:

- Medical device-compliant cryovials with capacities between 0.5 and 50 mL. Standard screw cap cryovials are not accepted as medical devices and should not be used for clinical materials (15, 108, 109).
- Bags with or without overwrap bags with volumes from 10 to over 100 mL. These are generally not suitable for volumes below 5 mL (15).
- Straws with capacities of up to 0.5 mL, originally developed for the cryopreservation of spermatozoa, oocytes and embryos. These straws can be efficiently heat sealed and have found some application with vitrified cell therapy products (110, 111).
- Syringes that can be cryopreserved and used for clinical delivery immediately on thawing are under development by a number of manufacturers and will be of significant value once commercially available (112, 113).

The need for innovative designs for freezing vessels will expand as therapies in regenerative medicine develop and will certainly be required for the cryopreservation of larger materials such as cell sheets and biomimetic tissues.

### Preparing the Final Sample

To ensure that the selected freezing containers can be filled easily and effectively this aspect of the process should be considered during protocol development. Manual filling of cryocontainers is possible when relatively small numbers of samples are involved but, for larger numbers, the impact of the lag time between dealing with the first and last sample must be evaluated. This lag may significantly extend the time that some preparations incubate in the cryoprotectant solution, compromising the viability and overall functional outcome of the product. With manual filling there may also be variability between samples bringing with it the possibility of inconsistent therapeutic effects. Automated filling lines are currently available for some vials and for straws but not, to date, for cryobags (15, 100).

Temperature monitoring and control during vessel filling are also important and influence the size of the batch that can be filled, as well as the conditions under which the operation takes place. Any potentially cytotoxic effects of cryoprotectants, especially DMSO, will be reduced by lowered temperature and the avoidance of abrupt temperature change. Consequently, working temperatures in the range 0–4°C are beneficial (15, 73, 75) and practices such as adding cooled protectant to precooled cells or warmer protectant to warmer cells with immediate subsequent cooling are to be recommended (114–116).

The cell concentration per unit volume of cryoprotectant medium can affect survival and this should also be determined during protocol development. Conventional concentrations employed for the cryopreservation of hematopoietic stem cells for engraftment range from 20 to 80 × 10^6^ nucleated cells/mL (117–119). Sample volume reduction, achieved by increasing cell concentration, can be attractive as it will limit materials and reagents used, processing time and cryostorage space as well as reducing the quantity of DMSO infused to the patient (11, 117–119). However, cell concentrations higher than ~200 × 10^6^ cells/mL appear detrimental on engraftment prediction (through the CFU-GM assay) or engraftment yield post-thaw (88, 114, 117, 118, 120, 121). As the ice fraction within a cooling sample grows and the unfrozen channels, where cells are confined, reduce in size there will be an increase in potentially injurious compression forces and direct cell to cell contact (11). This report also notes the increased likelihood of cell clumping, both before freezing and after thawing, as a result of using too high cell concentrations. By contrast, new immune cell therapies are reported to contain up to 10^7^ CAR-T cells/mL (122, 123) and low cell concentrations <10^6^ cells/mL can lead to cell apoptosis (124). A balance therefore needs to be established between using a greater injected volume at low cell concentrations and any cell damage caused by using high cell concentrations to achieve equivalent perfused cell numbers. The adopted concentration will also be affected by the percentage survival expected following cryopreservation and the total number of cells that need to be delivered into the patient. These considerations will also influence the choice of freezing container.

The possibility that a proportion of the cells may adhere to the freezing vessel walls during the cryopreservation process has also to be considered and the consequent level of recovery assessed. Also of concern is the sedimentation rate of suspended cells and cell aggregates in the cryoprotectant solution. Rapid sedimentation can dramatically change the cell concentration at the base of a cryovial, for example, and can have a significant effect on the immediate cell environment as freezing progresses through the bulk sample. The elapsed time between vessels being loaded with a cell suspension and ice nucleation, when cells begin to be immobilized, must be considered where cell concentration may have a discernible impact on post-thaw outcomes. The timing needs to be consistent between vials to minimize sedimentation-related effects, with minimal variation (125).

Of particular concern and relevance to cell therapy is the cryopreservation of samples containing a range of different cell types. Sensitivity to the cryopreservation process may be different between cell types and could result in subpopulation changes in the recovered material. In PBMC samples, T cells (CD3+/CD4+ and CD3+/CD8+ cells) are often identified as more sensitive than the other cell subpopulations (29, 126, 127), although a recent study outlined the greater sensitivity of Natural Killer cells (CD56+ cells) over T cells (CD3+/CD4+ and CD3+/CD8+ cells) in cryopreserved donor lymphocyte infusions (128). The sensitivity of T cells to cryopreservation may even be different within specific T cell subsets, with regulatory T cells (Tregs) and activated T cells generally more sensitive than other subsets (127, 129). However, allowing a post-thaw resting period before staining cells for flow cytometry analysis has been shown to improve the detection and functionality of PBMC subpopulations and T cell subsets. This resting period may allow for the repair of some aspects of cell injury, yet the nature and duration of such a recovery period has yet to be determined, and must be consistent between all samples, as does the time between sample staining and data acquisition (127, 129–133). This recovery time has been considered as enabling both the removal of apoptotic cells generated by the cryopreservation process and the pre-activation of T cells (131). An interesting observation is that the apparent sensitivity of T cells to cryopreservation relative to other PBMC subpopulations may be limited by the inclusion of a non-permeable CPA to the cryoprotective solution, in addition to DMSO (90, 134). This sensitivity also appears to decrease after a second freeze-thaw cycle (127), suggesting that there may be a subpopulation of cryotolerant T cells in the original sample. Cell damage experienced by dendritic cells (DCs) may also be different from the other PBMCs, and seem to be best cryopreserved with a combination of DMSO and a non-permeable sugar at a cell density of 10^7^ cells/mL (135).

For cell populations such as pancreatic islets and HSC, the initial cell preparation and extraction (before cryopreservation) can have a major impact on cryopreservation outcome. An inverse correlation has been reported between HSC viability and neutrophil contamination, therefore a poor collection can lead to a poor cryopreservation outcome and poor engraftment success regardless of cryopreservation strategy used (121, 132). A similar situation exists for pancreatic islet cryopreservation in which isolation of islets can result in losing up to half of the cellular material and it is likely that poor cryopreservation outcomes here are at least in part due to the sub-optimal condition of the surviving cells from the collection (136). In cases where collections cannot be practicably improved by the cryobiologist, strategies such as replacing DMSO with other cryoprotectants, or conditioning cells pre- and post- cryopreservation in stabilizing media (137) are to be considered.

For some applications, encapsulation of cells or cells seeded onto scaffolds may be required prior to cryopreservation (138, 139). An example of the former is the cultivation of cell spheroids within alginate beads, which is part of the manufacturing process of many bioartificial tissues, including liver (BAL) (65, 138, 140, 141). Cell encapsulation may provide greater functional performance, but spheroids are also difficult to cryopreserve due to physical ice damage suffered by the spheroid and the protracted cell dehydration times necessary during cooling to accommodate the diffusion distances within the spheroids (59, 65). Larger aggregates of cells, perhaps included in alginate beads, complicate cryopreservation as varying diffusion distances, thermal gradients and limiting membrane permeabilities can be present, and this complexity increases when the size of candidate product is increased e.g., cells growing around a scaffold, either natural or artificial (142, 143). Cryopreservation of cells or spheroids attached to scaffolds or other types of 3D constructs to support cell proliferation for tissue engineering must consider both damage to the scaffold itself as well as cell detachment caused by contraction and expansion of the scaffold material during cooling and warming (144–146).

### Cooling Rate

The appropriate, optimal cooling rate must be selected to minimize cell losses during cryopreservation and aid the effectiveness of the prepared therapy. The severe cellular injuries that can occur during cooling are primarily a consequence of ice formation in the system, as noted above, and cooling rate is the key influencing factor over when and where ice forms. Having control over cooling rate provides a level of control over ice formation and, consequently, cell survival (6, 10, 11, 107).

For a system comprised of cells in an aqueous, suspending medium, a relatively slow cooling rate results in ice forming first in the bulk phase i.e., the suspending medium. As previously stated, solutes are excluded from the crystals of this extracellular ice, causing an increasing osmolarity of the residual, as yet unfrozen, suspending medium (11, 69, 72, 74). This imposes potentially fatal osmotic stresses on the cells, commonly known as “solution effects.” However, this risk has to be balanced against the benefits resulting from dehydration of the cells in response to this new osmotic situation. As cytoplasmic concentration increases the probability of unavoidable, fatal, intracellular ice formation diminishes. If the applied cooling rate is too rapid, the extent of this dehydration is reduced, and the probability of intracellular ice formation increases (6, 10, 72). If the rate is too slow then dehydration may even become excessive and cause injury. Other deleterious effects due to cryoprotectant toxicity may also become apparent.

The cooling rate producing optimal survival for a particular cell/suspending medium combination will reflect the point of balance between the positive and negative effects of slow and rapid cooling (72). Additional factors that can influence the positioning of the optimal cooling rate will include biological issues such as the type and origin the cell preparation, the cell density of the sample, cellular surface area to volume ratio and cell water permeability (6, 7, 147, 148). Properties of the suspending medium, the properties of the cryoprotectants employed and any measures taken to induce ice nucleation will also influence the optimal cooling rate (6, 7, 10, 73).

In mammalian systems, optimal cooling rates can range widely, from tens to hundreds of °C/min for human red blood cells depending on the composition of the cryoprotective medium used (80) and sperm samples depending on their animal origin (149–152). Much slower rates, from 0.5 to 0.1°C/min, are appropriate for oocytes and embryos (153–155), ovarian tissue (35), and liver spheroids (64). For a wide range of somatic cell suspensions, reported cooling rates of c. 1°C/min provide consistent, high levels of recovery (9, 66, 119). To achieve this rate for small cell volumes, characteristic of research investigations and routine laboratory procedures, there are simple, passive freezing devices that can be cooled in a −80°C freezer (15, 156). However, these are not appropriate for clinical applications, due to their low sample capacity and cooling rate inconsistencies that result from inadvertent interventions e.g., when the freezer is opened for unrelated reasons, or from suboptimal use e.g., when underloading the device or stacking several of them in the freezer when limited space is available (15). Precise and reproducible control of cooling rate is best achieved using programmable, conductive or convective controlled rate freezing equipment that is constructed to deal with relatively large sample volumes.

As cell clusters, spheroids, organoids, and tissues become of increasing, therapeutic importance it may be that slower cooling rates have to be employed. This is because in a cell mass the diffusion distance for water between the innermost cells and the bulk, external medium become significant and thermal transfer will be similarly affected (59). These size-related effects have the effect of slowing protective dehydration in response to the osmotic gradient generated by the presence of extracellular ice, as well as slowing the rate of heat transfer from the cell mass. Very slow cooling rates may therefore be necessary to allow for cell dehydration to be effective for all the aggregated cells. Inevitably, however, some cells will experience excessive osmotic stress during this extended incubation and innovative, protective steps will become essential to limit any likely injury (65, 157, 158).

Tissue cryopreservation has also been limited by structural tissue damage caused by ice crystals. Typically, techniques employed for these tissues are similar to those for cell suspensions, and future research and development is required to produce new protocols that better couple macrostructure preservation with high cell survival. However, considerable success with slow cooling can be achieved where macrostructure is not critical for function e.g., in the cryopreservation of thymus (159) and ovarian tissues (35, 160). Where preservation of macrostructure is central to tissue function, as for heart and skeletal muscle and kidney and liver tissues, ice crystals would be avoided if appropriate vitrification could be developed, and this may be the focus of much future investigation (63, 161).

### Ice Nucleation

**Figure 3:**
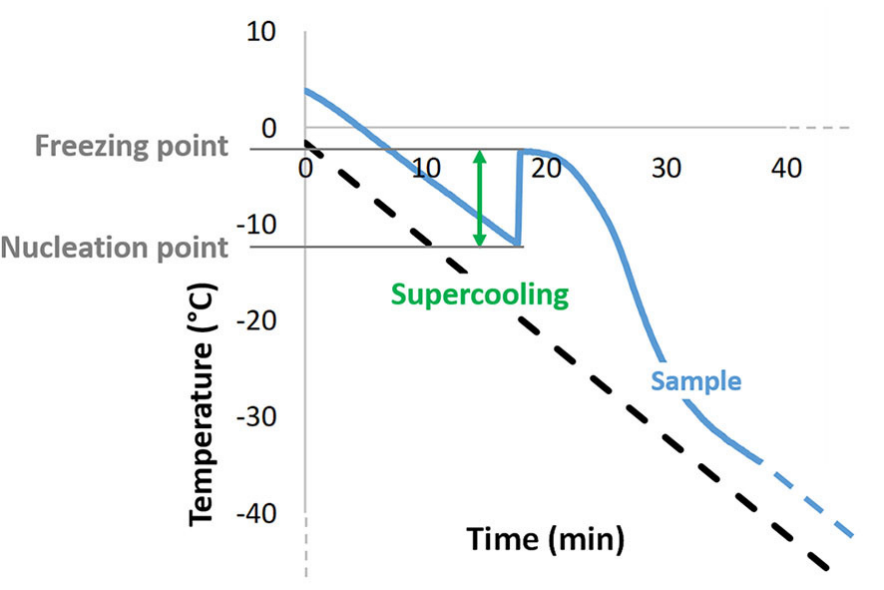
Typical temperature profile obtained during conventional, slow freezing of an aqueous sample, showing supercooling as the difference between ice nucleation temperature and the sample's freezing point, as well as the exothermic character of ice crystallization.

During continued, slow cooling ice crystals will form by nucleation at a temperature below their melting point (Figure 3), and aqueous solutions can cool significantly below this before nucleation and relatively rapid, crystal formation occurs (162, 163). This effect is known as supercooling and the point at which it ends can be detected by a rise in temperature (the ice exotherm) as the first ice crystals form in the bulk solution. This is due to the latent heat of fusion that is released during the transition of liquid water to crystalline ice (162, 164). The formation of ice crystals in a cell suspension may then impose some, or all, of the injurious stress on the cells that have been described above. The greater the extent of supercooling, the greater the immediate, stresses experienced by the suspended cells when the ice begins to form. To optimize a cryopreservation protocol, it is essential to understand the impact of this ice nucleation on cell recovery, and to consider if external interventions to induce it at the earliest, possible opportunity might be of value.

**Figure 4:**
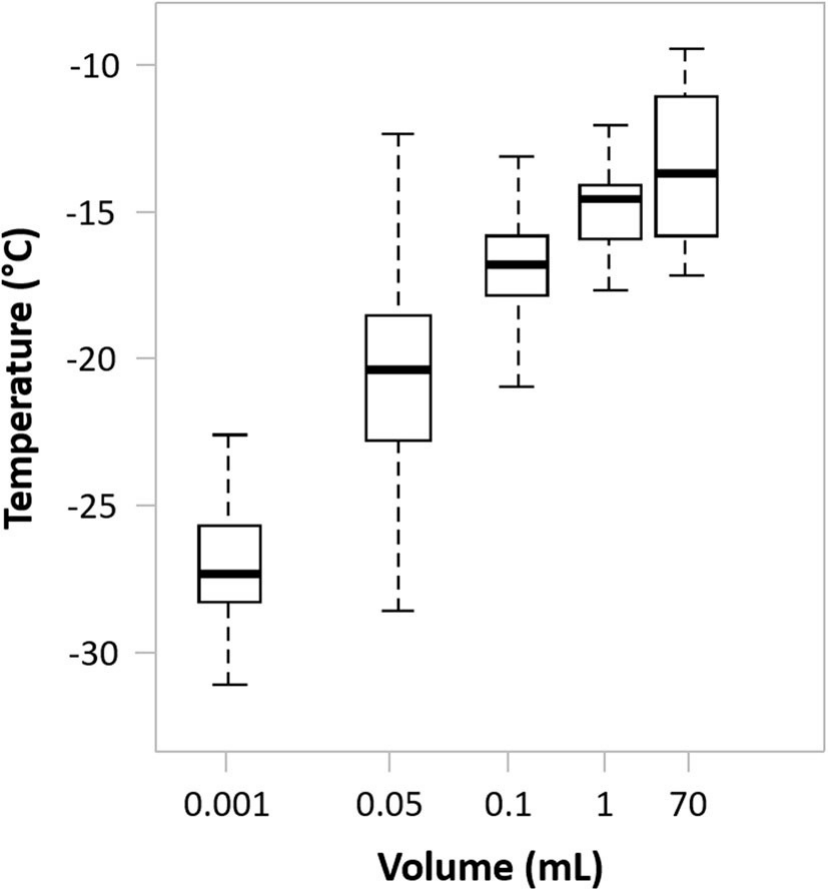
The effect of sample volume on spontaneous ice nucleation in a typical cell suspending medium. Drawn using internal data as well as data from Daily et al. (165).

Sample volume has a significant effect on ice nucleation, with the potential for supercooling increasing as sample volume decreases (Figure 4). For small volumes, typical of *in vitro* fertilization straws, multi-well-plates and small cryovials, delayed nucleation can cause extensive cell injury and so induced nucleation at the earliest possible, subzero temperature is often necessary (35, 162). This can be achieved by techniques such as mechanical agitation of the cooled samples, inducing a small, ultra-cold spot on the outer wall surface of the sample vessel wall or the use of additives (162).

However, cell therapy products are often cryopreserved in larger volumes (cryobags or large cryovials (from 10 mL up to 150–200 mL). Fortuitously, spontaneous ice nucleation for these volumes will occur at a temperature significantly closer to the melting point than would be the case for smaller volumes (Figure 4). Consequently, a limited, damaging effect is achieved without further intervention (165), although practical studies investigating the impact of induced nucleation for these larger sample volumes are lacking.

If nucleation were to be induced in these larger sample volumes, ice nucleating agents could be included in the samples as a practical method of choice. These agents act as catalytic templates for nucleation and subsequent crystal formation during cooling. Examples include cholesterol, silver nitrate, extracted bacterial proteins (166) or inert particulate minerals (12, 165). A major advantage of such agents is that they require no user input beyond their addition to the sample container, and compatibility with regulatory requirements and the desired clinical application, including patient contact must be ensured. Nucleation could also be induced by ultrasound waves passed through samples, triggering ice formation in a consistent and user-independent way (165).

There are also cryopreservation protocols that induce nucleation by the inclusion of a “plunge” or “seeding dip,” programmed in as part of the controlled cooling profile. At an appropriate point, close to the melting point of the suspending medium, a very rapid, limited cooling excursion is included in the protocol to lower the sample temperature by up to 10°C before returning to the original level. However, any beneficial impact of such a practice over conventional, linear cooling protocols has yet to be demonstrated (163).

## Handling, Extended Storage, and Transport

Following controlled rate freezing the cryopreserved product must be placed in controlled frozen storage. Care must be taken during transfer of the newly frozen sample from the controlled rate freezer to the storage/transport container as only a few seconds of warming may be all that is needed to compromise viability (167–169). There may be no visible change in the sample during a brief exposure to an elevated temperature, apparently indicating no change in the ice, yet melting at the microscopic level will start to occur at temperatures as low as approximately −50°C (170, 171). These, apparently subtle, changes can have a negative effect on the eventual, thawed recovery of the samples (164). For practical convenience, cooling to a minimum of −60°C is recommended before immediate and very rapid transfer to the storage freezer, to limit damage due to uncontrolled rewarming (170). If a little time is needed to ensure an efficient and safe transfer, then a pre-cooled container containing LN or dry ice can act as an interim holding container.

The minimum, safe storage temperature for the sample must be maintained, without deviation, throughout the cryochain to avoid any warming or thawing that could lead to viability loss or weakened function of the product. For maximum stability in this respect samples should be stored in the vapor phase immediately above LN (below −150°C) or in an ultra-cold freezer at temperatures below −130°C (10). Storage in bulk LN (−196°C) should be avoided in clinical situations as there is a real risk of microbial contamination, particularly in vessels that are accessed regularly and have been in use for an extended period (172–174). Further, to avoid cross contamination samples should not be stored with others known to pose a potential contamination risk in this respect.

The required shelf life for cryopreserved cell therapy materials will be dependent on application with, commonly, up to 1 year for authorized CAR T cell products (122, 123) and much longer for other, banked cell therapy products (11, 66, 97). Whilst storage at −80°C may be appealing as storage freezers at this temperature are standard laboratory items, this will not provide the stable level of recovery generally required (141, 175). In the special case of biobanks, where required storage periods are considered in at least decades, the extreme chemical stability secured by storage below the glass transition temperature of the cryoprotectant employed (c. −120°C for DMSO) is essential (10, 11, 170, 176).

Also central to effective storage are physical security of the storage facility, clear, and permanent labeling of samples (to be legible if frosting occurs readily) and a location plan for samples within the storage vessel. During cryostorage, care must also be taken not to warm other samples that may have to be moved to provide access to the material being located. Best practice will also employ alarms and continual temperature monitoring to prevent sample loss in the event of power failure or malfunction (172, 177, 178). Containers can be purchased with barcodes, or these, and other forms of identification, can be added as required. All access to the containers, for whatever reason, must be recorded. Robust sample identification ensures traceability and prevents errors in administration. It should become a matter of routine to trace the sample from tissue isolation to patient delivery, with sufficient security to protect patient confidentiality (179).

Frozen products should be transported in specialist containers that maintain the low temperature of their contained storage space using either LN trapped in a fibrous matrix within the container walls (dry shipper), or electrically cooled systems. Dry ice use should be restricted to very short transit of samples, e.g., from controlled-rate freezer to cryogenic storage, as some molecular mobility—albeit extremely slow—that can lead to sample deterioration can occur between the glass transition temperature of the sample and −80°C (170). Shippers are relatively expensive, durable items and the expectation is that they will be returned to sender for further use. However, the hospital or other facility accepting the frozen cells may not have a dedicated LN storage facility and will use the dry shipper as a temporary storage unit. For this to be effective there must be access to LN to top up the dry shipper as required, and an understanding of how frequently this may be required. Suitable, LN-free, portable devices for transport and subsequent storage at the recipient facility are also available, allowing frozen cells to be moved directly from the controlled rate freezer to a LN-free unit for shipping to, and storage at, the point of final use. Continuous temperature logging during storage and transportation is essential to the security of the cryochain and the accepting facility should be able to access this data up to the point of administration to the patient. Equipment to achieve this is available. Operation in this way should secure a tightly linked cryochain that can provide safe, multiple movement of frozen samples from initial tissue isolation to delivery of prepared, therapeutic materials to the patient (180).

It is important to note that because of the various contamination risks associated with LN-cooled equipment (172–174), the provider of the frozen therapy may have opted to use a LN-free controlled rate freezer for sample processing. If the storage, transport or recipient facilities revert to using LN-cooled containers then the hitherto clean cryochain will be compromised.

## Thawing and Recovery

For successful cryopreservation, the influence of cooling rate on the required thawing rate for optimal cell survival has to be acknowledged, as these are co-dependent processes. Relatively rapid cooling will result in the amount of extracellular ice in the frozen product being lower than that predicted by the equilibrium phase diagram for the system (69) (see also “section Cooling rate” above). Consequently, to prevent potential injuries due to further ice crystallization during thawing a relatively rapid rate of warming is required. This minimizes the level of hypertonic stress the cells will experience as thawing progresses, with a positive effect on survival (107). Where slower cooling rates are employed, as is typical for cell therapies in larger volumes e.g., in cryobags, the proportion of extracellular ice will be greater and will be closer to the equilibrium point. Slower rates of warming can be employed with a reduced risk of ice recrystallization and reduced osmotic stress during thawing (69, 107, 181, 182).

In regenerative medicine the widely accepted procedure used to thaw frozen cryobags has developed, with little modification, from the essentially subjective procedure used in basic research laboratories. Most recommended methods require the immediate transfer of the frozen sample from cryogenic storage into a water bath at 37°C, where it should be gently agitated without removal from the water. It is important that this transfer is made as rapidly as possible as a cryobag removed from its cryogenic environment will warm rapidly in air with a potentially major detrimental impact on post-thaw viability (164) (see also “section Handling, Extended Storage, and Transport” above). The final thawing process is accepted as complete at the point where the very last visible ice melts away. At this point the bag must be removed from the water bath to minimize the risk of overheating the sample (77). This subjective end point may vary between operators but in the hands of an experienced person, with a full understanding of what is required, there is no doubt that the procedure produces consistent and successful results. However, if the operator lacks familiarity and understanding of the process there is a risk of unacceptably variable recovery of viable cells, with reduced post-transplant performance (29, 183). With the increased use of cryopreserved materials, it is increasingly likely that thawing may be carried out by clinical staff with limited training or experience in cryopreservation. This possibility has to be positively managed to avoid a real risk to the clinical success of the therapy.

The presence in clean rooms or operating theaters of open water baths for warming presents an obvious contamination hazard as well as operational difficulties (102–104). These include maintaining the required water temperature, replacing evaporative water loss, avoiding microbial contamination and regular calibration to ensure accurate temperature control. Programmable equipment is becoming available to avoid such difficulties and provide the automated thawing of cryopreserved cell samples. This equipment will provide computer control of warming, enclosed thawing chambers and eliminate liquid warming media. The facilities for automatic calibration, data logging and data transmission to aid traceability and monitoring are also available. The use of this type of equipment should eliminate the subjective assessment of thawing and provide a consistent procedure that requires no specific expertise on the part of the operator. In addition to cryobags (48, 184, 185), non-cellular therapeutic materials such as plasma samples can be thawed using this type of equipment (186).

Once thawed there may be a need for further manipulation of a cell product has been before clinical use. This can include diluting the cells (perhaps within their cryocontainer), for immediate injection, washing the cell suspension to remove cryoprotectant before delivery or loading into a delivery vessel such as a syringe. However, there may be regulatory and practical constraints as to where and how this may be done, which may limit delivery of a treatment only to sites with appropriate culture facilities. Therefore, the development of cryopreservation protocols that avoid post-thaw manipulation can carry significant benefits.

It is essential that an immediate post-thaw assessment of viability is made to confirm the probable effectiveness of the delivered therapy. Cells should be thawed only as required and not maintained for any extended period of time before use, as viability can decline due to the inherent toxicity of the cryoprotectants. This difficulty may be reduced if the protocol being followed includes a post-thaw wash to dilute the cryoprotectants. When this is not included then thawed cells should be maintained for the shortest possible time at low (non-freezing) temperatures e.g., 4°C. The viability test must, of necessity, provide rapid results and should not require more than easily accessible, immediate laboratory support. Strong candidates for such a test are those that rely upon dye exclusion from cells e.g., trypan blue, indicating outer cell membrane integrity, and determined by simple light microscopy (187). However, these tests do not indicate viability in terms of coordinated cell structure and function and it essential that the positive correlation between the dye exclusion results and genuine cell functional recovery is understood. Such a correlation has been demonstrated for apheresis samples, where it is evident that dye exclusion consistently overestimates cell viability (188, 189). However, once such a relationship has been determined and understood the dye exclusion can be used as good indicator of eventual cell recovery in the administered therapy. Similar constraints apply to slightly more complex, immediate tests, such as the detection of fluorescein released from fluorescein diacetate by active esterase enzymes contained within an intact outer membrane. Viability assays which use these types of stain are non-specific and do not detect apoptotic or destroyed cells, which may be generated as a result of the cryopreservation process. Overlooking them may give a misleading picture of the state of a sample post-thaw, and co-staining strategies of each specific cell type of interest together with Annexin V and propidium iodide may be of value (78, 190). Finally, combining several types of assays (membrane integrity assay/enzymatic assay/functional assay) is recommended to achieve a comprehensive assessment of preservation efficacy (191).

To fully understand the possible effectiveness of an administered therapy it is also important to understand the likely reduction in viable, thawed cells in the hours or days after thawing. During this critical period, it is probable that some cell damage is repaired but there will be other structural or functional injuries, not readily identifiable, that are not repairable and may lead to cell death. In some instances, cryopreservation can also activate apoptotic pathways (192–194). The cell number contributing to the required therapeutic effect is, therefore, going to be lower than the estimated immediate post-thaw viability. The extent of such losses is an important piece of information when calculating dosage. These post-thaw effects argue strongly for the retention of sufficient quality control material from individual, cryopreserved samples so that incontrovertible viability tests, such as cell colony formation can be carried out, at different post-thaw time points. This information will contribute to ensuring the effectiveness of the therapy and inform discussions over the need for protocol optimization or modification.

## Regulatory Issues

Cell therapy materials are likely to be controlled by enforceable regulations where they are intended for routine treatment. The requirements likely to be needed for the approval of a regenerative medicine therapy should be considered during the development phase of the product. Where cryopreservation is involved in the preparation and/or storage of such materials, then regulations will probably also apply. The details may vary between national and international bodies, but quality control and traceability will be key elements of any effective, regulatory procedure. Common themes to be routinely considered will include sample sterility, reproducibility, efficacy, traceability, and the safety of cryoprotectants.

For example, the sample containers employed in the study should be capable of being hermetically sealed, favoring closed systems as much as possible to reduce contamination risks to a minimum (195), and the cryoprotectants and medium components should also be approved. Final delivery to the patient should also be considered for if the regulations prevent cryoprotectants being infused directly into the patient then a washing step will be required, with possible risks to post-thaw cell viability and performance. It is important that complete details of the cryopreservation process, including storage, transport, and thawing are tracked, recorded and retained together with the details of the samples and their history. This information must be held in a secure, and yet accessible, way.

Validation of processes is an important aspect to consider, both for regulatory issues and for ensuring that optimal cooling processes established are in fact followed during the cryopreservation process. For example, cryobags' temperatures will typically lag behind the programmed temperature of a controlled rate freezer—this is an inevitable consequence of the time taken for heat to flow out of a cryocontainer and larger volume (64, 107). It should be ensured that the key parameters such as cooling rate over the critical temperature range [i.e., from ice nucleation to intracellular glass transition (170)] are followed even when a temperature lag is taken into account. Often “dummy” cryocontainers are used—for example a cryovial of equal type and cryoprotectant fill but without cells—complete with a thermocouple to validate appropriate stages of the cooling process (12). Re-validation of a process is critical when any aspect of the process (for example volume, cryocontainer, or freezing/cooling device) is altered.

Quality control (QC) samples are commonly retained for use before cell therapy treatment and are also an important, contributory part of effective regulation. Consequently, it is important to ensure that the QC sample has a cryopreservation history that is as close as possible to that of the bulk therapy. This can pose practical challenges as, typically, the QC sample is of significantly lower volume than the bulk sample. During freezing, even when frozen together, there may be a significant difference in rate of heat loss and degree of supercooling before ice nucleation between the two volumes. This can produce differences in their post-thaw survival (133, 163). Recovery of the smaller, QC sample may therefore not accurately reflect the outcome when the bulk sample is thawed and it is essential to be aware of, and compensate for, such issues. Recent developments considering “mini-bags” as opposed to vials or segments of tubing as is currently most common may present a solution to these QC challenges (196).

## Future Direction

Effective cryopreservation and an efficiently managed cryochain are becoming increasingly recognized as providing a key enabling technology to support the delivery of successful cell therapies to the patient. The recent and ongoing development of CAR T cell therapies and the increasing administration of dendritic and mesenchymal stromal cells (13, 197) argue for increased employment of cryopreservation in collection centers, CT manufacturing sites and clinical settings. This is an important change as allogeneic hematopoietic stem cell transplantations were traditionally carried out using fresh grafts, just after collection from a related or unrelated donor (14). The ability to handle larger quantities of material and to provide suitable cryogenic storage and shipment solutions are necessary to ensure an effective cryochain is available to link the laboratory to the clinical front line. The recent recommendation from hematopoietic transplant networks worldwide to cryopreserve all stem cell products ahead of allogeneic transplantation (26–28, 198, 199) is a strong argument in support of development in this direction. During a pandemic, cryopreservation could provide additional time to assess whether a cell donor carries a disease such as Covid-19 before a graft is infused, as well as increasing flexibility in the transplantation process to provide a more streamlined experience for donors, patients and healthcare systems (14, 198–200).

Despite extensive experience in using cryopreserved products for autologous stem cell transplantation there are concerns regarding the limited amount of published data confirming that cryopreserved allogeneic stem cell products achieve equivalent outcomes to freshly infused ones (200). It has been pointed out that where cryopreservation cannot be performed at the collection site or where transport delays or travel restrictions unpredictably delay cryopreservation (27, 200), there is an enhanced risk of reduction of cell viability and engraftment success when the sample is eventually cryopreserved (200, 201). It has to be accepted that deterioration of cell function will begin immediately after sample collection and will continue up to the time of cryopreservation. This highlights the importance of managing clinical situations, where possible, to provide for cryopreservation at the same site and time as sample collection. In this way a secure and efficient cryochain all the way from the donor to the patient can be provided.

For the continuing development of effective cell therapies in regenerative medicine there is a parallel, continuing need to develop new cryopreservation protocols. These are likely to be based on relatively large cell volumes of suspended cells in cryobags or similar containers, and must meet the clinical requirements, and handling practicalities, for efficient delivery to the patient. For this type of material, relatively slow cooling rates together with relatively slow warming will be employed. To be as effective as possible, the details of recommended protocols will need to be aligned as closely as is practicable to the specific requirements of the cell types concerned, leading to a growth in studies of optimized processing. The design of innovative protocols will also become increasingly necessary as new types of cell materials become important and available.

The successful cryopreservation of larger volumes, as in engineered constructs (>1 mm in any dimension) is also going to become increasingly important. This will include cells attached to fabricated structures, spheroids, and organoids, where problem areas include increased diffusion distances that affect the transfer of heat and cryoprotectants. There may also be issues related to mechanical damage, caused by ice crystals, that disrupts the cell-to-cell contacts that are an essential feature of a functioning construct. Exceptions do exist, however, as with ovarian and thymus material where tissue architecture is less important for cellular function.

The valuable contribution that cryopreservation can make to regenerative medicine will be accelerated, and enhanced, by the interchange of information between clinical teams and those involved in basic research and development. Importantly, this dialog will contribute to ensuring a good understanding of the process by all those involved in operating and maintaining effective cryochains.

## Author Contributions

GM conceptualized and reviewed the work. JM and PK worked on curation, analysis and visualization of the data presented, prepared the original draft, edited it and reviewed it, and contributed equally to this work. All authors contributed to the article and approved the submitted version.

## Conflict of Interest

All authors are employees of Cytiva, Danaher Corporation, and work in the development of cryopreservation devices and strategies. No Cytiva equipment or reagents are mentioned in this review.
