# Supplementary material for: Cryopreservation as a Key Element in the Successful Delivery of Cell-Based Therapies—A Review
Source: Front Med (Lausanne). 2020 Nov 26;7:592242. doi: 10.3389/fmed.2020.592242 (PMC7727450; doi:10.3389/fmed.2020.592242)
Supplement: Supplementary file 1 [file Data_Sheet_1.docx]

Improving recovery of cryopreserved material in regenerative medicine by a systematic investigation of the complete cryochain

This investigative outline aims at giving practical advice regarding each point of the cryochain to be considered when improved recovery of cryopreserved material for regenerative medicine is sought after. The procedures set out below are supported by the information and further references provided in the main body of the associated, published review.

When the clinical team has concerns over the performance of cryopreserved material administered during cell therapy, an investigation of the procedure may be required. This should take the form of a critical analysis of each step in the cryochain, starting at the viable cell count from the initial tissue isolate to the point of delivery to the patient, as outlined in the flow diagram presented in Supplementary Figure 1. The goal will be to achieve an optimised protocol that delivers the maximum number of viable cells into the cryochain after freezing and retains the maximum possible number of these cells after thawing and recovery. It is important to view the cryopreservation protocol as extending beyond the freezing step and not ending until the material has been thawed and recovered for use. The potentially high numbers of viable cells provided by optimised freezing can be dramatically eroded by suboptimal events up to, and including, post-thaw recovery.

To ensure such an investigation is maximally effective the issues and concerns that initiated the inquiry from the clinical perspective should be shared with a specialist in cryopreservation. This will help to shape and conduct the practical investigations appropriately and can provide a gateway to necessary laboratory facilities and wider cryobiological experience.

# Viability Testing

Establishing a primary base-line value for viable cell number (VCN) is the essential first step in an investigation to secure an optimised cryopreservation protocol. This first data-point, against which all others will be compared, should be determined for the prepared cells/tissue and immediately before treatment with any cryoprotectant agent (CPA) would begin.

For practical purposes the viability testing should be as simple and rapid as possible, and scoring cells that exclude dye e.g. trypan blue, examined by light microscopy, meets these criteria. To be clear, however, dye exclusion indicates outer cell membrane integrity but does not indicate viability in terms of normal cell structure or co-ordinated function. Similar limitations apply to assays such as observing cells treated with fluorescein diacetate that retain the fluorescein released by active esterase enzymes. This assay only provides information on membrane integrity and the activity of a single enzyme. As well as microscopy-based assays, viability can also be assessed using flow cytometry. Whichever assay is used it is important that it has been calibrated for the cells in question against an assay that reflects whole cell recovery competence, such as colony formation.

References:

Van Buskirk RG. Viability and functional assays used to assess preservation efficacy: the multiple endpoint/tier approach. In: Baust JG, Baust JM, editors. Advances in Biopreservation. 1st ed. CRC Press; 2006. p. 123–43.

Robb KP, Fitzgerald JC, Barry F, Viswanathan S. Mesenchymal stromal cell therapy: progress in manufacturing and assessments of potency. Cytotherapy. 2019 Mar 1;21(3):289–306.

Barcelo H, Faul J, Crimmins E, Thyagarajan B. A practical cryopreservation and staining protocol for immunophenotyping in population studies. Curr Protoc Cytom. 2018 Apr;84(1):e35.

# The Sample Before Freezing

A first, and straightforward, expedient to improve the inadequate effectiveness of cryopreserved product may be to increase the cell numbers in the sample to be frozen. Many regenerative medicine applications require a large number of cells and an optimum cell concentration for successful cryopreservation has been reported. Tracking viable cell number /mL immediately after thawing will allow for comparison with the required cell count for clinical effectiveness. It is important to note that survival after cryopreservation can be diminished if the cell concentration of the sample is too high. Cell sedimentation that may occur in small cryovials before freezing should be avoided, by gentle agitation, as this can mimic excessive cell concentrations in the lower portion of the vial. Cell concentration and size are also major considerations when cryopreserving cell spheroids and organoids and, typically, the greater the number of cells per colony the lower the post thaw outcome.

References:

Alencar S, Garnica M, Luiz RR, Nogueira CM, Borojevic R, Maiolino A, et al. Cryopreservation of peripheral blood stem cell: the influence of cell concentration on cellular and hematopoietic recovery. Transfusion (Paris). 2010;50(11):2402–12.

Félix OMW de O, Tunes G, Ginani VC, Simões PC, Barros DP, Delbuono E, et al. The influence of cell concentration at cryopreservation on neutrophil engraftment after autologous peripheral blood stem cell transplantation. Hematol Transfus Cell Ther. 2018;40(3):233–9.

Kung YK, Cobos E, Morgan D, Park M, Dixon S, Wu K, et al. High cellular concentration of peripheral blood progenitor cells during cryopreservation adversely affects CFU-GM but not hematopoietic recovery. J Hematother. 1996 Feb;5(1):73–7.

Kilbride PJ. Experimental and mathematical modelling of the culture and cryopreservation of a bioartificial liver device utilizing a 3D cell scaffold construction [Doctoral dissertation]. UCL (University College London) 2016. 350 p.

# Locating Sub-Optimal Steps in the Cryochain

The following steps will locate problem areas within the cryochain where unacceptable loss of viability occurs. Resolution of any discovered issues will require further, detailed research effort.

A cautionary note:

Where the availability of material is limited, many of these necessary investigations may be carried out using relatively small volumes of cells e.g. in 2mL cryovials. The sample volumes within these may not respond to cooling in the same way as in larger containers (e.g. medical cryobags) due to differences in thermal diffusion distances, the thermal transfer properties of the construction materials involved, as well as in the degree of supercooling due to differing ice nucleation temperature probabilities. Precise detail of the cooling and warming profile of samples can be monitored by a recording temperature probe placed directly into a dummy sample. Attaching the temperature probe to the outer surface of a sample is a good alternative as inner probes may act as inducers of ice nucleation, and as the temperature difference between each side of the container wall is minimal. Adjustments to the freezer programme can then be made to ensure that the cooling histories for both smaller and larger containers are as required, and comparable.

Reference:

Kilbride P, Meneghel J, Lamb S, Morris J, Pouzet J, Jurgielewicz M, et al. Recovery and post-thaw assessment of human umbilical cord blood cryopreserved as quality control segments and bulk samples. Biol Blood Marrow Transplant J Am Soc Blood Marrow Transplant. 2019 Sep 6;25(12):2447-53.

## 3.1 Comparison of cell viability before and after CPA treatment to identify problems at this early stage of the procedure.

It is probable that the protocol under investigation is based upon DMSO as the cryoprotectant, from 5% v/v being a good starting point as a trade-off between cryoprotective efficacy and limited cytotoxicity, up to the widely used 10%. Altering this concentration should be considered together with the possible inclusion of other permeating, or non-permeating, protectants. Reducing the concentration of DMSO, or its complete replacement, may be considered at this stage if there are indications that it may not be ideal for specific, clinical circumstances. The temperature and duration of the incubation in the CPA-containing medium, prior to freezing, should also be investigated. The addition of antioxidants and anti-apoptosis agents may be of value.

References:

Bahsoun S, Coopman K, Akam EC. The impact of cryopreservation on bone marrow-derived mesenchymal stem cells: a systematic review. J Transl Med. 2019 Nov 29;17(1):397.

Barcelo H, Faul J, Crimmins E, Thyagarajan B. A practical cryopreservation and staining protocol for immunophenotyping in population studies. Curr Protoc Cytom. 2018 Apr;84(1):e35.

Worsham DN, Reems J-A, Szczepiorkowski ZM, McKenna DH, Leemhuis T, Mathew AJ, et al. Clinical methods of cryopreservation for donor lymphocyte infusions vary in their ability to preserve functional T-cell subpopulations. Transfusion (Paris). 2017;57(6):1555–65.

Weng L, Beauchesne PR. Dimethyl sulfoxide-free cryopreservation for cell therapy: A review. Cryobiology. 2020 Jun;94:9–17.

## 3.2 Comparison of cell viability before and immediately after freezing to identify problems within the freeze-thaw steps of the protocol

It is important to locate, precisely, where in the freezing protocol the bulk of viability is being lost. The first step is to compare viability after CPA treatment with viability after ice nucleation. This can be achieved by following the freezing protocol and thawing cells from -20°C, where it can be safely assumed ice has formed in the container. The solution to a problem identified at this step would be to take control of nucleation and introduce a step modification to induce it at the earliest opportunity.

The second step in this study would be to thaw cells from the point at which they are transferred from the CRF to liquid nitrogen (LN), with exposure to LN for a brief period e.g. 1h. Difficulties recorded here would indicate that the ice nucleation point should be further investigated and that the controlled cooling rate to the transfer point might need to be adjusted. The nucleation point is identified by the rise in sample temperature (the latent heat exotherm) recorded as ice forms.

Manual induction of ice can be difficult in many CRFs and so the use of additives, or ultrasound, should be considered. Helpfully, the spontaneous nucleation point of typical cell suspensions in cryobags is relatively close to ice melting point and so nucleation problems are likely to be limited. In any investigation of nucleation, it must be borne in mind that, without induction, a small volume of cell suspension in a cryotube will reach a lower temperature than a similar, bulk suspension in a cryobag before ice forms.

Published literature, to date, concerning the samples being considered here indicate that the optimal cooling rate from 4°C to -60°C will not deviate greatly from 1°C min^-1^ so it is unlikely to be the root cause of significant viability reduction.

References

Morris GJ, Acton E. Controlled ice nucleation in cryopreservation – A review. Cryobiology. 2013 Apr 1;66(2):85–92.

Daily MI, Whale TF, Partanen R, Harrison AD, Kilbride P, Lamb S, et al. Cryopreservation of primary cultures of mammalian somatic cells in 96-well plates benefits from control of ice nucleation. Cryobiology. 2020 Apr;93:62–9.

## 3.3 The post-thaw viability on immediate recovery from LN should be compared to viability after cryogenic storage. Any significant reduction of viability at this step will indicate problems with the handling of frozen material and/or storage conditions.

One of the central advantages of cryopreservation is that, once safely frozen and stored, there will be no chemical changes within the cells that can compromise viability or performance. This holds for storage time measured in tens of years if the storage temperature remains below the glass transition temperature of the cryoprotectant employed (c. -120°C for DMSO). Once it is confirmed that viable cells, in acceptable number, have been cryopreserved then the rest of the cryochain must be precisely managed to ensure that this viability is not diminished.

If a frozen sample is inadvertently exposed to temperatures above the glass transition temperature, then changes can occur that will compromise viability following eventual thawing. These changes need not be as extreme as any melting of ice but changes that can occur in the conformation of the frozen medium during an elevated temperature excursion can have adverse effects on the pattern of eventual ice melting. Moving samples within a storage vessel to improve access to other materials is common, as is transfer between vessels to facilitate transport. Unless carefully done events such as these can be the cause of unacceptable viability loss. To minimise these risks, and make visible potential issues, it is essential that monitoring systems are in place so that each access to a storage vessel is logged, together with the task performed. Clear labelling of samples and a location plan for samples within the storage vessel are essential for security. Also, data recorders should accompany cryopreserved materials in transit so that an acceptable temperature history can be confirmed by the recipient. Where samples are stored in the shipping vessel, once delivered and before use, it is essential that the sample temperature is monitored up to the point of thawing.

References:

Meneghel J, Kilbride P, Morris JG, Fonseca F. Physical events occurring during the cryopreservation of immortalized human T cells. PLoS One. 2019 May 23;14(5):e0217304.

Massie I, Selden C, Hodgson H, Fuller B. Storage temperatures for cold-chain delivery in cell therapy: a study of alginate-encapsulated liver cell spheroids stored at -80°C or -170°C for up to 1 year. Tissue Eng Part C Methods. 2013 Mar;19(3):189–95.

Angel S, von Briesen H, Oh Y-J, Baller MK, Zimmermann H, Germann A. Toward optimal cryopreservation and storage for achievement of high cell recovery and maintenance of cell viability and T cell functionality. Biopreservation Biobanking. 2016 Oct 28;14(6):539–47.

## 3.4 Sample thawing

For successful thawing, conventional practice requires the immediate transfer of the frozen sample from cryogenic storage into a water bath at 37^o^C. Care must be taken to ensure that this transfer takes no more than a few seconds, as the frozen sample will warm rapidly in air leading to the possibility of potentially lethal injury and increased variability in the product quality post-thaw. The value for comparison is the viability recorded after initial transfer to cryostorage.

Monitoring of the procedure should ensure that the sample remains in the bath, with gentle agitation, until the moment the last ice melts, and no longer. Once thawing is complete, delay in removing the sample from the bath can result in damaging overheating. The moment of completed thawing is essentially subjective and, where possible, the same trained and experienced operator should be responsible for determining this, and a tightly defined standard operating procedure is required. In the correct hands, and with appropriate facilities, the water bath thawing procedure will produce consistent results. Programmable equipment for the automated thawing of cryopreserved cell samples is becoming available, providing computer control of warming together with facilities for automatic calibration, data logging and data transmission. Adopting this type of equipment should ensure a consistent procedure that requires no specific expertise on the part of the operator.

As the optimum warming rate is coupled to the cooling rate employed in the protocol, there is little scope for manipulation of thawing conditions to improve cell recovery without considering revision of much of the rest of the protocol. If full compliance with the thawing requirements is still followed by unacceptable cell recovery, then it is possible that injury is being caused elsewhere in the protocol but takes some time to become evident (see below).

References:

Baboo J, Kilbride P, Delahaye M, Milne S, Fonseca F, Blanco M, et al. The impact of varying cooling and thawing rates on the quality of cryopreserved human peripheral blood T cells. Sci Rep. 2019 Mar 4;9(1):3417.

Baust JM, Campbell LH, Harbell JW. Best practices for cryopreserving, thawing, recovering, and assessing cells. In Vitro Cell Dev Biol Anim. 2017 Dec;53(10):855–71.

## 3.5 Cell injury and repair after thawing

Monitoring of cell viability after thawing may reveal a delayed, unacceptable loss of viable cells, below the immediate post-thaw level. This can indicate problems with the freeze/thaw protocol and subsequent investigation should look for delayed mortality after each step of the protocol, to locate the problem area. Toxic responses to cryoprotectant may be involved in post -thaw mortality and it is important to ascertain if a rinsing step is necessary to remove or dilute the protectants, and how quickly after thawing this should be done. The rate of dilution of the protectant during rinsing must also be considered as there will be an optimum speed to avoid osmotic shock to the cells.

This type of viability loss may reflect an inherent sensitivity of the cells to the process. The resolution to a problem of this kind is to look to significant modification within the protocol. Even in the most effective protocols it is probable that processes of repair will be necessary for some cells after thawing and it may be that an *in vitro* recovery period before clinical use will be required.

References:

Baust JM, Vogel MJ, Snyder KK, Van Buskirk RG, Baust JG. Activation of mitochondrial-associated apoptosis contributes to cryopreservation failure. Cell Preserv Technol. 2007 Sep 1;5(3):155–64.

Woods EJ, Thirumala S, Badhe-Buchanan SS, Clarke D, Mathew AJ. Off the shelf cellular therapeutics: Factors to consider during cryopreservation and storage of human cells for clinical use. Cytotherapy. 2016 Jun 1;18(6):697–711.

**Supplementary Figure 1 Legend.** Flow diagram to aid in the development or improvement of a successful, cGMP-compliant cryopreservation protocol of biological material in regenerative medicine applications, where oval, lozenge and rectangular shapes represent terminators, decisions and processes, respectively (T: temperature, CRF: controlled rate freezer, DMSO: dimethyl sulfoxide, CM: culture medium, CPA: cryoprotective agent, LT: long term, PT: post-thaw, cGMP: current good manufacturing practices).
