# Supplementary figures and images for: Cryopreservation as a Key Element in the Successful Delivery of Cell-Based Therapies—A Review
Source: Front Med (Lausanne). 2020 Nov 26;7:592242. doi: 10.3389/fmed.2020.592242 (PMC7727450; doi:10.3389/fmed.2020.592242)

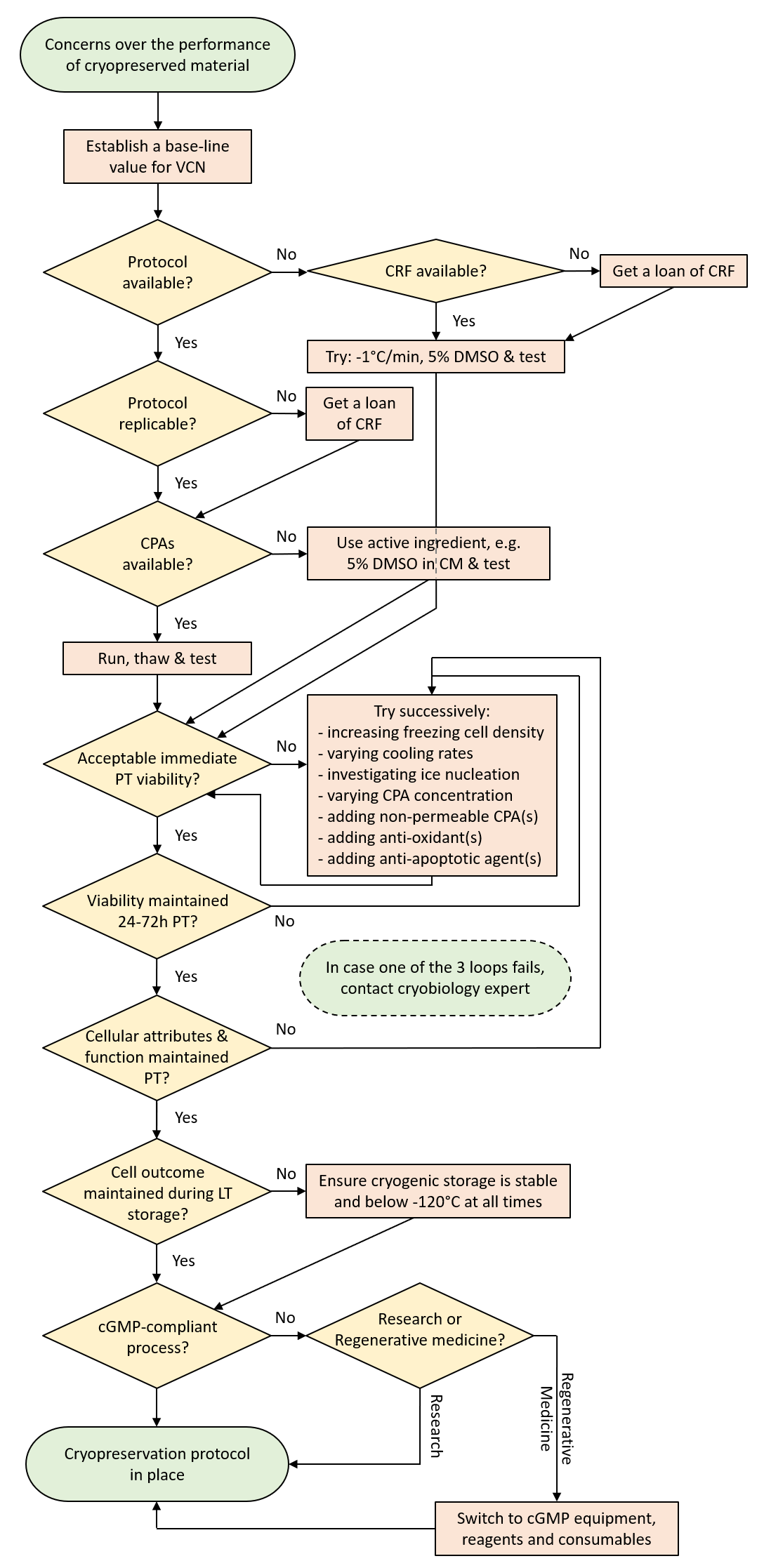

Supplement: Supplementary file 2 [file Image_1.tif]
